# Development of a comprehensive flourishing intervention to promote mental health using an e-Delphi technique

**DOI:** 10.3389/fpsyt.2023.1064137

**Published:** 2023-02-17

**Authors:** Juliane Piasseschi de Bernardin Gonçalves, Camilla Casaletti Braghetta, Willyane de Andrade Alvarenga, Clarice Gorenstein, Giancarlo Lucchetti, Homero Vallada

**Affiliations:** ^1^Department of Psychiatry, School of Medicine, University of São Paulo, São Paulo, Brazil; ^2^Nursing Department, Santo Agostinho University, Teresina, Brazil; ^3^Institute and Department of Psychiatry (LIM-23), University of São Paulo Medical School, São Paulo, Brazil; ^4^Department of Pharmacology, Institute of Biomedical Sciences University of São Paulo, São Paulo, Brazil; ^5^Department of Medicine, School of Medicine, University of Juiz de Fora, Juiz de Fora, Brazil

**Keywords:** depressive symptoms, flourishing, mental health, intervention protocol, positive psychology, e-Delphi technique

## Abstract

**Background:**

Although observational studies have already shown promising results of flourishing, a broader concept of health based on positive psychology, there is still a gap in the literature regarding studies that combine different topics of flourishing in a single intervention.

**Objectives:**

To develop a comprehensive and integrate intervention based on positive psychology gathering different topics of flourishing to improve mental health outcomes in individuals with depressive symptoms.

**Methods:**

The following steps were performed: (1) a comprehensive literature review; (2) the designing of a 12-session group intervention based on the values, virtues, and topics of flourishing; (3) assessment of the rationale, coherence, and feasibility by a panel of healthcare professionals answering semi-structured questions, and (4) application of an e-Delphi technique including mental health experts to reach a consensus of at least 80% for each item of the protocol.

**Results:**

A total of 25 experts participated in the study, 8 in the panel with semi-structured questions and 17 in the e-Delphi technique. A three-round e-Delphi technique was required to reach a consensus for all items. In the first round, a consensus was reached for 86.2% of the items. The remaining items (13.8%) were either excluded or reformulated. In the second round, a consensus was not obtained on one item, which was reformulated and approved in the third round. Qualitative analyses of the open questions were performed and suggestions for the protocol were considered. The final version of the intervention was composed of 12 weekly group sessions with 90-min each. The topics included in the intervention were physical and mental health, virtues and character strengths, love, gratitude, kindness, volunteering, happiness, social support, family, friends and community, forgiveness, compassion, resilience, spirituality, purpose and meaning of life, imagining the “best possible future,” and flourishing.

**Conclusion:**

The flourishing intervention was successfully developed using an e-Delphi technique. The intervention is ready to be tested in an experimental study to verify its feasibility and effectiveness.

## 1. Introduction

The mental health burden is increasing worldwide, posing several challenges to low-to-middle income countries such as Brazil. The World Health Organization (WHO) estimates that more than 300 million people have depression worldwide, and less than half have access to treatment (1).

Depression is currently the most significant cause of absenteeism and disability in the workforce (2) and the second largest cause of disability worldwide.

The incidence of depression has increased by 59.8% from 1990 to 2017, and is the most common chronic disease worldwide, at present (3). Depression treatment costed approximately US$ 236 billion in 2018 in USA, an increase of more than 35% since 2010 (4). According to recent data, at least 14% of the Brazilian population suffers from depressive symptoms, while 17% have had an episode of major depression throughout their lives (5).

The WHO defines health as “a state of complete physical, mental and social wellbeing and not only the absence of disease” (6). However, this definition does not consider the dynamic nature of human beings. Therefore, new concepts are emerging in literature. We would like to highlight a specific one called flourishing, coined by Tyler VanderWeele, which defines health as “the state in which all aspects of a person's life are good” (7). According to this concept, there are five broad domains of human life: (i) happiness and life satisfaction; (ii) health, both mental and physical; (iii) meaning and purpose; (iv) character and virtue; and (v) close social relationships and four pathways, i.e., family, work, education, and religious community (8).

Previous longitudinal studies with large samples have already shown promising effects of VanderWeele's flourishing dimensions/pathways on physical and mental health. A US national cohort with ~13,000 adults aged >50 years has shown that individuals with greater purpose of life had lower mortality risk (9) and that altruistic behaviors (e.g., volunteering) were associated with lower mortality, and better physical activity and psychosocial outcomes (10). On similar lines, a US cohort with ~60,000 nurses found that forgiveness was associated with higher levels of positive affect and social integration and lower levels of psychological distress (11), and religious attendance was related to lower risk of death (12).

This longitudinal data supports that the broader concept of health has robust scientific evidence and should be discussed by the scientific literature. Within this context, several interventions based on human values and virtues were proposed, and publications on positive mental health programs (13, 14) showed promising results toward minimizing depressive symptoms in different populations (15–18). Although there are several clinical trials regarding the effectiveness of virtues, values, and character on health outcomes (19, 20), to the best of our knowledge, there is a scarcity of studies combining different aspects of flourishing, that focus on VanderWeele's concept (8). Since the concept of flourishing embraces different dimensions which are linked to better health outcomes, promoting all of these dimensions in a single intervention may have better results than considering them separately.

The purpose of this study was to advance this field of research by creating an intervention protocol combining different aspects of flourishing to deliver in a few group sessions. Therefore, this study aimed to develop a simple, practical, and low-cost intervention protocol to promote mental health based on the conceptual framework of flourishing, through an e-Delphi technique.

## 2. Materials and methods

The study was approved by the Research Ethics Committee of the School of Medicine of the University of Sao Paulo, Brazil, under approval number CAAE: 52554221.4.0000.0068. All respondents provided written informed consent.

The study was organized into four phases, as shown in Supplementary Figure 1.

### 2.1. Phase 1: Comprehensive literature review

A non-systematic review was carried out in the following scientific databases: PubMed, Web of Science and Scopus, using the keywords “Flourish^*^,” “Mental Health,” “Positive Psychology,” “Intervention,” “Therapy,” and “Treatment.” We selected higher hierarchy evidence studies, such as meta-analyses performed through systematic reviews, randomized clinical trials, and cohort studies.

First, we selected studies that investigated the impact of the five domains of Tyler VanderWeele's concept of flourishing, as presented in the Introduction: (i) happiness and life satisfaction; (ii) physical and mental health; (iii) purpose and meaning of life; (iv) character and virtues, and (v) social relationship. Then, we elected the specific topics that integrate these domains.

### 2.2. Phase 2: Session design

The most relevant topics related to flourishing were selected from these studies and included in our intervention. We created the structure of the intervention based on the protocols used by the clinical trial and adopted a model with the aim of gaining complexity and deepening the themes at each session. In this phase, we also defined the specific objectives to guide the group discussion within the theme of the session, and the specific dynamics and exercises related to the theme, based on previously published clinical trials.

### 2.3. Phase 3: Panel with semi structured questions

The first version of the protocol was submitted to a committee of healthcare professionals, experts in the field of mental health, with experience in leading therapeutical groups or clinical practice related to spirituality and religiosity, for a non-systematic evaluation of its feasibility, robustness, and coherence, using a set of semi-structured questions. An email was sent to the experts explaining the flourishing intervention and inviting them to assess the attached protocol through an electronic questionnaire. After agreeing with the Informed Consent Form (ICF), they evaluated the protocol using a semi-structured questionnaire with open-ended questions about the intervention structure: content, format, target population, and intervention providers. The results obtained from the experts were used to remodel the protocol. Based on the modifications suggested by the experts, the protocol was then prepared for the structured e-Delphi phase.

### 2.4. Phase 4: Structured assessment through the e-Delphi technique

In this phase, another group of experts (different from Phase 3) were invited to assess the intervention through an e-Delphi technique. This type of systematic methodology allows receiving opinions and comments from a panel of selected experts (21, 22). Thus, the opinion of experts on the subject can help point out possible topics of the intervention that need improvement or are inadequate. When the experts' answers are quantified, it serves as a guidance on the appropriateness of the evaluated item. If there is no consensus among the experts, the item should be changed or withdrawn (22).

Health professionals from medical and non-medical fields were included; they were different from those invited in the previous phase. The selection criteria were PhD holders with at least 10 years of experience in any of the following areas: mental health, spirituality/religiosity, positive psychology, or complementary health therapies. These professionals were selected based on their academic and clinical experience on themes related to the development of values and virtues in clinical practice and research, mental health care toward depressive patients and use of complementary therapies. Since there is no consensus on the sample size of experts needed for a panel adequate for an e-Delphi technique (23), the present study determined to include at least 17 individuals (22). This choice was based on the findings of a previous systematic review that investigated the median number of panel members among 76 published protocols.

Questionnaires were developed on the SurveyMonkey^®^ platform and the link was sent by email. Experts had 45 days to respond. The e-Delphi technique was expected to be conducted for as many rounds as necessary until consensus was achieved for all items.

The questionnaire consisted of general questions about each session and specific ones for the items that were considered the most challenging by the authors. It contained multiple choice questions and open questions for extra comments. The answers were structured with a Likert score of 1 to 5 points. Although no consensus is defined for the e-Delphi evaluation criteria, most studies use levels of agreement between 60 and 80% (24). Therefore, to be rigorous with the assessment of the protocol, we adopted a cut-off point of 80% consensus with scores between 4 and 5 points. In the case of not reaching the cut-off point, an item could be eliminated or re-assessed.

Comments by the evaluators were examined, and the results of this analysis were used to improve the protocol and materials of the intervention.

## 3. Results

### 3.1. Phase 1 and 2: Comprehensive literature review and session design

The themes of the intervention's sessions were derived using the conceptual framework and the activities for Flourishing proposed by Tyler VanderWeele and collaborators in a previous publication (25). The selected topics of flourishing were: physical and mental health awareness; purpose and meaning of life; forgiveness; character strengths and virtues; kindness; volunteering; spirituality; gratitude; imagining the “best possible future”; love; compassion; social support, family, friends, and community; and happiness and resilience (25).

**Figure 1:**
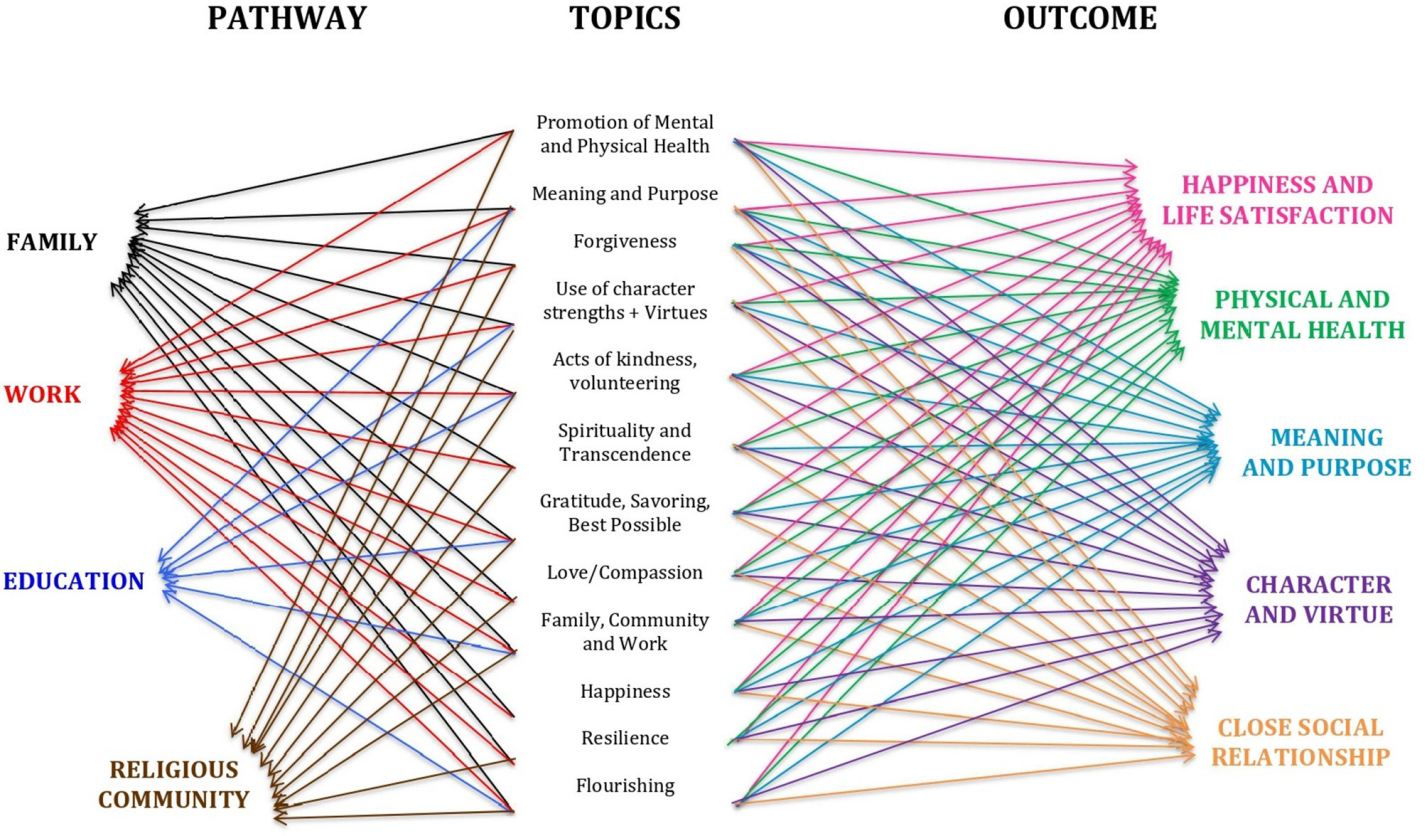
Flourishing framework: The pathway interconnections between the domains and the aspects.

The framework in Figure 1 was developed to illustrate the interconnection between the pathways of flourishing, the topics selected for the intervention sessions, and domains of flourishing, demonstrating the rational used to develop the entire intervention protocol.

Thereafter, we categorized the topics into three main groups to increase the complexity and enhance “human development” in the process of flourishing:

a) Health awareness and self-knowledge: Physical and mental health, virtues, character strengths, and flourishing.
b) Virtues and values that can connect the participant with the environment and others: Kindness, volunteering, gratitude, imagining the “best possible future,” love, compassion, and social support, family, friends, and community.
c) Virtues and values that connect one with self: Happiness, purpose and meaning of life, forgiveness, spirituality, and resilience.

Regarding the structural organization of the sessions, our search revealed that most positive psychology interventions varied from 1 to 20 weekly sessions (19, 20), with a duration of 1–5 h per session (19). Therefore, in accordance with the literature, we decided to distribute the topics into 12 weekly sessions, each lasting for 90 min.

Also, the intervention was to be delivered on-line. Online interventions can be effective and feasible for treating mental health disorders, such as depression and anxiety (26, 27). Advantages of such interventions include adaptability, multimedia presentations, and saving on traveling efforts. Due to these reasons and considering the traffic problems as a consequence of the population density and territorial extension of the city of São Paulo, Brazil, we decided to offer the intervention through the online format. Regarding the structure of the intervention, the sessions were to be conducted by one or two healthcare professionals where all participants could interact with the provider and among themselves.

Table 1 presents the topics in the sequential order of the initially proposed approach, goals for each session, and specific tools used to achieve the goals. All sessions aimed to promote individual reflection using different strategies such as, group discussions, writing exercises, guided meditation exercises, sharing videos and songs about the topic of the sessions, and reflective moments. Based on the theoretical model, we chose to describe the evidence found for each topic along with the design and goals proposed for the session.

**Table 1:** Preliminary protocol sessions, objectives, and strategies used in each session.

**Session**	**Objectives**	**Strategies used**
1. Promotion of physical and mental health/presentation of the intervention	(a) Discuss health concepts (b) Impact of flourishing on depression	• Video about health and attitudes• Group discussions about health and lifestyle • Awareness exercises for changing lifestyle
2. Gratitude and imagining a “better future”	(a) Identify reasons for gratitude in life (b) Plan the best possible future scenarios	• Video about gratitude and empathy• Group discussions about gratiful attitude future plan • Writing exercises about gratitude and the best future
3. Love and compassion	(a) Reflect on the concepts of love and compassion (b) Stimulating acts of compassion and self-compassion	• Writing exercises about compassion acts • Guided mental visualizations about loving attitudes and compassion • Group discussions about behavior and health concerning compassion
4. Acts of kindness and volunteering	(a) Reflect on acts of kindness and their own attitudes (b) Stimulate engagement in volunteering	• Video about kind attitude and volunteering • Group discussions about how to be kinder• Awareness exercises about volunteering in feasible manners
5. Happiness	(a) Reflect on happiness(b) Encourage the construction of paths that involve happiness	• Guided mental visualization to identify happiness • Video audio of a lyric about the meaning of happiness • Group discussions about how to improve happiness in life• Writing exercises about attitudes regarding happiness
6. Family and friends/community	(a) Identify and discuss the social support network (b) Stimulate growth and improvement of the identified network	• Awareness exercises about the current network • Video audio of a lyric about friendship • Group discussions on how to improve social support
7. Forgiveness	(a) Identify reasons to forgive(b) Raise awareness about willingness and attitude of forgiveness (c) Reframe negative/challenging experiences	• Writing exercises about the will to forgive and strategies for accomplishing forgiveness • Group discussions about intentions and how to overcome pain
8. Resilience	(a) Identify and raise awareness of the process of recovering from negative experiences (b) Reframe negative/challenging experiences(c) Raise awareness of problem flexibility	• Writing exercises about challenges in life and coping mechanisms • Group discussions about facing problems and reframing them • Awareness exercises about resilience strategies
9. Character strengths and virtues	(a) Reflect on self-perception of one's own virtues(b) Stimulate the development of latent virtues and reinforce the present/identified ones	• Group discussions about the participant's virtues • Awareness exercises to strengthen their character
10. Spirituality and transcendence	(a) Reflect on spirituality and coping (b) Stimulating a life that incorporates more spiritual aspects for the individual	• Group discussions about beliefs and spiritual vision in life • Awareness exercises to improve spiritual coping mechanisms
11. Meaning and purpose of life	(a) Stimulate the search for a meaning in life(b) Ponder on the purposes of one's own existence	• Group text reading about meaning in life • Writing exercises for identifying meaning • Group discussions about how to act on a purpose in life
12. Flourishing and closing the intervention	(a) Integrate all the concepts and virtues discussed (b) Flourish a number of aspects at the end of the program(c) Reinforce exercises for the aspects that made the most sense for the participant	• Group text reading about team effort • Group discussions about the interconnection of the sessions • Awareness exercises on how to develop and grow as a human being

#### 3.1.1. Session 1: Physical and mental health

There are different interventions to raise awareness of physical and mental health (28, 29), and evidence shows that higher awareness helps in seeking and adhering to the treatment. The objective of this session was to discuss different health concepts and ways to flourish with depressive symptoms (8, 30). Providers would help participants understand different health concepts and reflect on their attitudes and behaviors toward their own health through video and group discussions.

#### 3.1.2. Session 2: Gratitude and imagining your “better future”

Previous evidence based on a meta-analysis showed that gratitude interventions were effective to improve individuals' psychological wellbeing and gratitude levels but not anxiety (31). In this context, writing lists of the things or people one is grateful for and writing letters to the people/situations were associated with better outcomes (32). Likewise, envisioning the “best possible future” was another effective strategy compared to other motivational techniques, as observed in a previous meta-analysis (33).

The goal of this session was to encourage the participants to identify situations in their lives that they were grateful for. We proposed watching a reflexive video and writing exercises regarding gratitude and plans for a “better future.”

#### 3.1.3. Session 3: Love and compassion

A meta-analysis of clinical trials on self-compassion showed a significant improvement in 11 psychosocial outcomes when compared to the controls (34). The focus on compassion, however, showed a small effect size in another study (35). Loving-kindness interventions (e.g., meditation) showed a small to large effect size in daily positive emotions when compared to other groups (35). This session aimed to help the participants identify acts and feelings of love and compassion in their daily lives through guided imagery exercises for compassion and writing exercises with group discussions on loving attitudes.

#### 3.1.4. Session 4: Kindness and volunteering

Regarding kindness and altruism, a recent meta-analysis pointed out a small effect size on wellbeing (36). Another meta-analysis evidenced a reduction in mortality for individuals engaged in some voluntary work (37). This evidence motivated the development of this session that aimed to make participants aware of the influence of kindness and volunteering on their health, using video and discussions about acts of kindness and volunteering. The provider would guide the group to create new, simple, and practical ideas about volunteering in everyday life.

#### 3.1.5. Session 5: Happiness

There is solid evidence that happiness is associated with lower mortality and better mental health outcomes (38). Based on previous intervention protocols that motivated participants to find “passion” (39, 40) and identify sources of happiness (41), the main goal of this session was to help participants reflect on how they could achieve a happier and healthier life, by making a list of what brought happiness to their lives, sharing these experiences with the group, and listening to music about the topic.

#### 3.1.6. Session 6: Family, friends, and community

Longitudinal studies have shown that having family, friends, and a relationship are associated with a better quality of life (8, 42). Likewise, there are clinical trials proposing strategies (e.g., positive psychology) to improve relationships (43, 44) and stimulating social support networks (45, 46) with positive results.

Thus, this session was designed for the participants to identify their social support network and improve the quality and quantity of their relationships. Providers would use open-questions and a song to raise awareness of participants' current network and discuss how to expand it.

#### 3.1.7. Session 7: Forgiveness

A meta-analysis evaluated the effectiveness of psychotherapeutic interventions to promote forgiveness, showing reduced levels of depression and anxiety and higher hope when compared to other treatments (47). Similarly, another meta-analysis showed that having empathy for the offender and overcoming feelings of unforgiveness were associated with lower levels of depression and anxiety (48).

Since most clinical trials use writing exercises to stimulate intention and feelings of forgiveness (49–51), we developed the session based on writing exercises to explore the participants' feelings and thoughts about forgiveness, identify reasons to forgive, and its impact on their health.

#### 3.1.8. Session 8: Resilience

A meta-analysis showed that resilience is negatively associated with negative mental health outcomes and positively associated with positive outcomes (52). Clinical trials proposed interventions based on cognitive-behavioral exercises and mindfulness (53, 54). We based the design of this session on Steinhardt and Dolbier's model of (a) transforming stress into resilience, (b) taking responsibility, (c) focusing on empowerment interpretations, and (d) creating meaningful connections (55).

The resilience session aimed to identify the process of recovering from adversities and developing a more flexible vision of those adversities. Providers would stimulate strategies to develop better resilience in life.

#### 3.1.9. Session 9: Strengths of character and virtues

A meta-analysis of character strengths interventions showed significant increases in positive affect, happiness, and life satisfaction, and lower levels of depression (56). In this context, the classic intervention proposed by Seligman (57) showed that encouraging individuals to exercise their strongest character strengths weekly, after identifying them through a questionnaire, resulted in increased happiness and decreased depressive symptoms.

We designed the session using an abbreviated version of the Seligman Strengths and Character scale for the participants to identify their strongest and weakest virtues. The main objective was to increase the participants' perception of their virtues and reinforce their strengths by sharing different perspectives.

#### 3.1.10. Session 10: Spirituality

Several studies have demonstrated the positive impact of spirituality-based interventions on mental health outcomes, such as lower levels of anxiety and depression (58, 59). Protocols are usually based on motivational group discussions addressing topics such as faith, spiritual beliefs, and peace (60, 61).

The session was based on the material developed by Hopkins et al. (62), with the objective to reflect on the influence of the individuals' beliefs on health by discussing the history of their belief system, and their connection with the sacred, others, and nature. Finally, providers would help participants on how to use these tools and spiritual practice to develop a healthier path.

#### 3.1.11. Session 11: Meaning and purpose of life

Meta-analyses on the meaning of life and its impact on health have shown positive correlation with life satisfaction and negative correlation with negative affect (63, 64). Likewise, there is growing evidence that clinical trials based on meaning-centered therapies are associated with better psychological outcomes (65).

This session was developed based on an intervention proposed by Luz et al. which used different reflexive texts about meaning of life (66), allowing participants to identify their meaning in life and consider a purpose to follow. Providers would help participants with their process of identifying and creating life purposes and being able to live it by proposing discussion from the reading of a text and exchange of impressions and personal experiences.

#### 3.1.12. Session 12: Flourishing

Finally, the aim of the last session was to integrate the virtues and values of the aspects of flourishing into participants' life, reinforcing the idea of human development. The session would encourage the participants to reflect on their role in life and assume responsibility for their lives and situations through text reading and group discussions (25, 67, 68).

### 3.2. Phase 3: Panel with semi structured questions

The panel was composed of eight experts: five women (71.4%) and three men (28.6%). Their professions were psychologists (four), physicians (two), social worker (one), and spiritual counselor (one), and all had more than 10 years of professional experience.

According to the experts, the intervention seemed to be a positive, innovative, and well-grounded proposal. They pointed out the following strengths: the scope of the themes, potential to stimulate reflection, and possibility of being replicated in different scenarios. The experts agreed with the feasibility of the intervention, emphasizing clarity and objectivity, and the applicability of the exercises. The number and duration of the sessions were considered appropriate. Most experts agreed that the providers should be healthcare professionals with previous adequate training on the subject and the sessions.

According to the experts, the population that could benefit from this intervention were adults and older adults with mild and moderate depressive symptoms. However, they discouraged the use of this intervention for individuals with severe symptoms. Furthermore, experts mentioned that individuals with low education could benefit from the intervention as well. However, they pointed out two possible challenges to be tested in practice: the writing exercises and the difficulties in understanding complex concepts such as resilience and flourishing. Finally, they reported that some conceptual adaptations and simplifications would be necessary.

Based on the opinions of this panel, adaptations were made in the protocol and the revised version was sent to an e-Delphi panel.

### 3.3. Phase 4: Structured assessment through an e-Delphi technique

Of the 21 experts invited, 3 were not available to collaborate in due time, and 1 refused to provide consent to participate. Therefore, the final panel was composed of 17 individuals: 10 men (58.8%) and 7 women (41.2%). The average age was 52.2 (SD = 14.5) years, with an average professional experience of 25.9 (SD = 10.6) years, and all of them were PhD holders. The panel included eight physicians, four psychologists, three university professors, one physical therapist, and one nurse.

This phase was separated as follows:

(a) E-Delphi technique assessment: This included 58 items regarding specific opinions on the objectives, strategies, and interventions for each session, using a Likert scale ranging from 1 (totally disagree) to 5 (totally agree).

*1*^*st*^ round

Table 2 presents the specific items assessed if consensus was achieved for them, and the conduct provided by the authors. Consensus was obtained on 50 out of the 58 items in the first round, representing 86.2% agreement among experts; these topics and exercises were maintained in the intervention protocol. The remaining eight items (13.8%) were from the following topics: three items from session 2 “gratitude and imagining a better future,” and one item each from session 6 “family, friends, and community,” session 7 “forgiveness,” session 10 “spirituality,” and session 11 “meaning and purpose of life.” All of these items were either excluded or reformulated and re-sent to the experts in the second e-Delphi round.

**Table 2:** Consensus for the items of the preliminary intervention protocol submitted to the Delphi technique.

**Evaluated items**	**First round**		**Second round**		**Third round**	
	**Consensus (%)**	**Conduct**	**Consensus (%)**	**Conduct**	**Consensus (%)**	**Conduct**
**Session 1: Promotion of physical and mental health/presentation of the intervention**						
1.1. How clear is the purpose of the session?	94.1	Maint.	–	–	–	–
1.2. How adequate is the dynamics of the initial presentation to the group?	88.2	Maint.	–	–	–	–
1.3. How adequate are the activities used to discuss the concept of health with the participants?	82.4	Maint.	–	–	–	–
**Session 2: Gratitude and imagining a “better future”**						
2.1. How clear is the purpose of the session?	**76.5**	**Revis**.	100	Maint.	–	–
2.2. How adequate is the exercise in which the participant is asked to name a historical figure they draw inspiration from?	**70.6**	**Exclu**.^*^	–	–	–	–
2.3. How appropriate is the exercise that encourages the participant to imagine a “bright and happy” future?	88.2	Maint.	–	–	–	–
2.4. How appropriate are the homework exercises?	**70.6**	**Exclu**.^*^	–	–	–	–
**Session 3: Love and compassion**						
3.1. How clear is the purpose of the session?	82.4	Maint.	–	–	–	–
3.2. How important is it to create a list of traits of loving/kind people the participant relates to?	94.1	Maint.	–	–	–	–
3.3. How important is it to promote love and compassion through guided mental visualizations?	88.2	Maint.	–	–	–	–
3.4. How important is it to discuss how the participant feels about guided mental imagery?	94.1	Maint.	–	–	–	–
3.5. How important is it for the participants to exercise love and compassion through the homework?	88.2	Maint.	–	–	–	–
3.6. How appropriate are the homework exercises?	82.4	Maint.	–	–	–	–
**Session 4: Acts of kindness and volunteering**						
4.1. How clear is the purpose of the session?	88.2	Maint.	–	–	–	–
4.2. How important is it for the participant to reflect on their experiences with volunteering?	94.1	Maint.	–	–	–	–
4.3. How important is it to encourage the participant to engage in volunteering activities in their community?	94.1	Maint.	–	–	–	–
4.4. How appropriate are the homework exercises?	100	Maint.	–	–	–	–
**Session 5: Happiness**						
5.1. How clear is the purpose of the session?	94.1	Maint.	–	–	–	–
5.2. How adequate is it to reflect on the participant's perception of happiness?	88.2	Maint.	–	–	–	–
5.3. How important is it to reflect on what makes the participant happy and what level of happiness they are currently experiencing?	100	Maint.	–	–	–	–
5.4. How important is the exercise addressing barriers and paths to happiness?	100	Maint.	–	–	–	–
5.5. How appropriate are the homework exercises?	94.1	Maint.	–	–	–	–
**Session 6: Family and friends/community**						
6.1. How clear is the purpose of the session?	88.2	Maint.	–	–	–	–
6.2. How important is it to discuss the meaning of family and friends with the participant?	88.2	Maint.	–	–	–	–
6.3. How appropriate is it to present the concept of social support to the group?	82.4	Maint.	–	–	–	–
6.4. How suitable is the song chosen by the research team for the session?	**70.6**	**Revis**.	82.3	Maint.	–	–
6.5. How appropriate is it to reflect on the changes the participant has made in his/her environment and relationships since the begining of the intervention?	82.4	Maint.	–	–	–	–
6.6. How important is it for the participant to count on his/her family to solve everyday problems?	94.1	Maint.	–	–	–	–
6.7. How appropriate are the homework exercises?	88.2	Maint.	–	–	–	–
**Session 7: Forgiveness**						
7.1. How clear is the purpose of the session?	100	Maint.	–	–	–	–
7.2. How appropriate is it to talk about situations that upset the participant and made them want to forgive?	94.1	Maint.	–	–	–	–
7.3. How important is it to mention potential benefits of forgiveness?	94.1	Maint.	–	–	–	–
7.4. How important is it to encourage the participant to change their perspective by putting themself in the shoes of the person who wronged them?	**76.5**	**Exclu**.^*^	–	–	–	–
7.5. How important is it to encourage the participant to put themself i the other people's shoes?	**70.6**	**Exclu**.^*^	–	–	–	–
7.6. How appropriate are the homework exercises?	88.2	Maint.	–	–	–	–
**Session 8: Resilience**						
8.1. How clear is the purpose of the session?	88.2	Maint.	–	–	–	–
8.2. How easy is it to understand the exercise that presents the phases of a problem to discuss resilience?	88.2	Maint.	–	–	–	–
8.3. How clear is the exercise that encourages the participant to look at the positive and negative aspects of a problem?	82.4	Maint.	–	–	–	–
8.4. How appropriate are the homework exercises?	82.4	Maint.	–	–	–	–
**Session 9: Character strengths and virtues**						
9.1. How clear is the purpose of the session?	100	Maint.	–	–	–	–
9.2. How adequate is it to present Seligman's Strength of Character Scale to the participant?	82.4	Maint.	–	–	–	–
9.3. How appropriate is it to ask the participant to name their strongest virtue and situations in which they had to apply it?	94.1	Maint.	–	–	—-	–
9.4. How appropriate are the homework exercises?	82.4	Maint.	–	–	–	–
**Session 10: Spirituality**						
10.1. How clear is the purpose of the session?	**76.5**	**Revis**.	**76.4**	**Revis**.	100	Maint.
10.2. How adequate is it for the participant to reflect on their own concept of spirituality?	82.4	Maint.	–	–	–	–
10.3. How appropriate is it for the participant to reflect on moments in their life when they clunge to or abandoned their spiritual beliefs?	82.4	Maint.	–	–	–	–
10.4. How appropriate is it for the participant to reflect on the role spirituality plays in their lives today?	94.1	Maint.	–	–	–	–
10.5. How appropriate are the homework exercises?	82.4	Maint.	–	–	–	–
**Session 11: Meaning and purpose of life**						
11.1. How clear is think the purpose of the session?	88.2	Maint.	–	–	–	–
11.2. How appropriate is the reading proposed for the session?	**64.7**	**Revis**.	82.3	Maint.	–	–
11.3. How important is it to discuss the participant's goals in life?	94.1	Maint.	–	–	–	–
11.4. How important is it to discuss the alignment between the participant's goals in life and their occupation?	88.2	Maint.	–	–	–	–
11.5. How appropriate are the homework exercises?	82.4	Maint.	–	–	–	–
**Session 12: Flourishing and closing the intervention**						
12.1. How clear is the purpose of the session?	82.4	Maint.	–	–	–	–
12.2. How adequate is the reading proposed in the session?	94.1	Maint.	–	–	–	–
12.3. How important is it for the participants to reflect on the activities carried out throughout the program?	100	Maint.	–	–	–	–
12.4. How helpful is it for the participants to reflect on the changes they experienced during the intervention program?	100	Maint.	–	–	–	–
12.5. How appropriate is the song chosen by the research team for the final session?	82.4	Maint.	–	–	–	–

Legend: Maint. = maintened item; Exclu.^*^ = excluded item; Revis. = revised item. The bold indicated to highlight all consensus items that did not reach at least 80%.

*2*^*nd*^ round

The authors considered the qualitative answers of the experts regarding the eight revised items and decided to exclude four items from the protocol and reformulate the other four to obtain consensus. Table 3 shows the summary of the experts' comments along with the authors' appropriate explanation for the reformulation or exclusion of the items. Only the “spirituality” objective of session 10 did not obtain a consensus and was revised again.

**Table 3:** Non-consensual items: Revisions and general comments.

**Evaluated items**	**Conduct**	**Summary of experts comments**
**Session 2: Gratitude and imagining a “better future”**		
2.1. How clear is the purpose of the session?	Revis.	Both topics were considered unrelated to what was discussed in the session
2.2. How adequate is the exercise in which the participant is asked to name a historical figure they draw inspiration from?	Exclu.^*^	The exercise at issue does not contribute much to the gratitude topic.
2.4. How appropriate are the homework exercises?	Exclu.^*^	Need to simplify the exercises with practical strategies so that the participants can continue improving after the program.
**Session 6: Family and friends/community**		
6.4. How suitable is the song chosen by the research team for the session?	Revis.	The song chosen could bring up feelings of sadness and nostalgia
**Session 7: Forgiveness**		
7.4. How important is it to encourage the participant to change their perspective by putting themself in the shoes of the person who wronged them?	Exclu.^*^	This specific exercise could awaken feelings of guilt or traumatic memories
7.5. How important is it to encourage the participant to put themself i the other people's shoes?	Exclu.^*^	This specific exercise could awaken feelings of guilt or traumatic memories
**Session 10: Spirituality**		
10.1. How clear is the purpose of the session?	Revis.	Participants may have religious or non-religious beliefs and experiences, so the objective of the session should be to embrace diversity/explore both belief systems
**Session 11: Meaning and purpose of life**		
11.2. How appropriate is the reading proposed for the session?	Revis.	The text focuses more on gratitude and the overcoming of obstacles than on an existential perspective

Revis. = revised item; Exclu.^*^ = excluded item.

*3*^*rd*^ round

The third round of the e-Delphi comprised a revised aim of the spirituality session. The experts' suggestions were mainly to highlight the aspects of spirituality related to transcendence, promote the meaning and experience of connection, and emphasize that the session goal should be more inclusive and cover religious and non-religious people. The item was reformulated, and the session renamed to broaden the complexity of the concept of spirituality adopted for the session. The item was then submitted for a third round of evaluation and was approved with 100% consensus.

The agreement for the ratings of all Delphi items between experts (inter-rater reliability) were assessed using the intra-class correlation coefficients (ICC) (two-way mixed model, consistency, average-measures ICC), yielding a coefficient of 0.48 (CI 95%: 0.25–0.66, *p* < 0.001).

(b) General protocol organizational opinions: This included 10 items concerning organizational aspects of the protocol, such as target public, number and duration of sessions, multimedia presentation, and the provider's guide, using multiple choice questions and Likert scale ranging from 1 (totally disagree) to 5 (totally agree).

**Figure 2:**
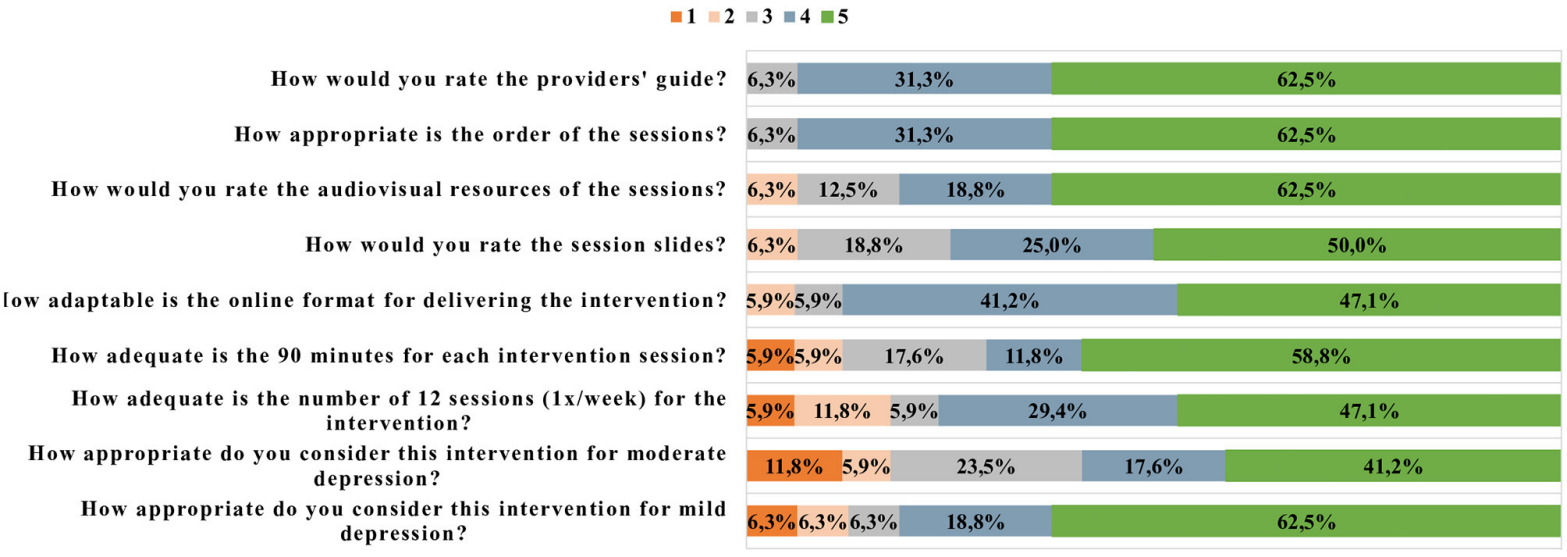
Experts' opinions about the structure and implementation of the protocol.

Figure 2 shows the opinions of the experts regarding the structure and implementation of the protocol. Experts highlighted the appropriateness of the overall process of flourishing, target population of those with mild depressive symptoms, 12 weekly online delivery sessions, use of the audiovisual resources, and the guideline created for the providers. However, some experts raised two points of warning: implementing the intervention for individuals with moderate depressive symptoms and duration of the sessions.

It is important to emphasize that changes were necessary to improve the coherence of the intervention as a whole. This included the changes related only to the structure (writing and distribution), and not to the content of the items, and the opinions on the e-Delphi technique were respected during this process. Another aspect that should be highlighted is that despite the reorganization of the themes, the protocol remained of 12 sessions.

(c) Experts' suggestions: Experts were allowed to suggest and comment in support of their opinions concerning the questions they were asked. They provided practical suggestions regarding group dynamics for the implementation of the protocol, that were included in the final manual created by the authors for the training of healthcare providers of the intervention in future.

The complementary exercises of the sessions were gathered in a single file, and it was decided to provide this material to the participants at the end of the intervention (post-intervention material), so that they could continue following the proposals and exercises in their daily lives, resulting in continuation of its flourishing process. Experts mentioned the importance of a strategy to ensure continuity of exercises for constant personal improvement, even after of the intervention ended.

## 4. Discussion

The present study successfully developed an intervention protocol based on the flourishing concept of health using a comprehensive literature review and the opinion of experts through the e-Delphi technique. The protocol consists of sharing therapeutic tools during online sessions in collaboration with trained healthcare professionals to conduct the sessions. Though there are other mental health programs focusing on positive aspects, but in this intervention, we specifically sough to adopt the VanderWeele's concept of flourishing and combine its various topics into the sessions offered (13, 14). This intervention is different from others because it combines all virtues and values in a single intervention, resulting in a more holistic and comprehensive approach.

For the development of this protocol, our study followed previous articles that used Delphi or e-Delphi to develop an intervention (21, 22, 69). Delphi is a technique that can impact ways of thinking or decision making through the convergence of opinions and comments of experts' assessments (21). The main advantage of using such a technique is the possibility of exploring underlying assumptions regarding a specific topic (69).

According to the literature, one of the most important aspects of Delphi is the choice of experts. Our experts were mostly from the field of mental health, and this choice was made considering the main outcome of our intervention (reducing depressive symptoms and promoting mental health) and the experience of the experts in the precepts of positive psychology. However, it is important to highlight that, to improve the public health feasibility of this protocol, professionals from other healthcare fields were also included. Another important choice for our panel selection was to include experienced PhD holders for the e-Delphi. This choice guaranteed that well-qualified experts with expertise in both clinical practice and research were included, which was supported by previous studies (21, 22).

An important choice in the development of our protocol was the use of an online e-Delphi technique, instead of a Delphi technique. The e-Delphi has some important advantages such as time and cost savings, convenience for experts and the research team, and data management facility (21, 22). The individualized communication with experts and their blindness regarding the other experts' answers during the process may have contributed to the impartiality of scores, ideas, and suggestions provided.

Regarding the protocol, the experts favored the online format of the interventions. The advantages of using this type of approach include the use of multimedia for the participants, facilitating access to complex content, time and cost savings, and the possibility of engaging with others (26, 27). The use of an online approach is supported by previous studies where clinical trials of brief online intervention programs showed better long-term clinical effects compared to face-to-face therapies for different mental health conditions, especially when the technique included the support of a healthcare professional (70).

As verified through the results, experts reached consensus for ~90% of the items, revealing that the intervention was consistent with the proposal of flourishing. However, it is important to highlight that one item was subjected to three rounds of e-Delphi to reach consensus: the objective of the spirituality session. To achieve the goal for this session the following concept mentioned by Puchalski (71) was adopted: “spirituality is the aspect of humanity that refers to the way individuals seek and express meaning and purpose and the way they experience their connectedness to the moment, to self, to others, to nature, and to the significant or sacred.” Some experts argued that spirituality should be related to the sacred or an immortal being and not to the connectedness to the moment, self, or others. Since our main goal in this session was to be inclusive, Puchalski's definition was chosen because it provides a more comprehensive understanding of this construct, in which individuals can recognize their beliefs and values, and identify which connections are meaningful to them, regardless of sacred or religious beliefs. Most experts highlighted the notions of transcendence and connection as relevant aspects to be addressed in this session. In the third round a consensus was reached and the session was renamed “Spirituality and Inner Connection.”

Another important highlight by the experts was the order of the topics of flourishing. The experts did not initially agree to address gratitude and imagining your “best future” in the same session, even though both topics could stimulate an individual to connect with the environment and with others. Comments included that the exercises proposed should be provided separately to achieve their personal goals, and that imagining a “better future” could be more useful at the end of the intervention to synthesize the concept of flourishing. The authors revisited the order of the topics maintaining the rationale initially proposed, to achieve an increase in complexity of the virtues and values of the process of flourishing (8).

Regarding the structure of the intervention, the experts agreed on the 12 weekly sessions; however, not all experts agreed with the duration of 90-min. Since online interventions have operational challenges, such as speed of connection and learning of operating the multimedia (70), a loss of therapeutic time should be considered. Also, group sessions including 10–15 participants need adequate time to allow everyone to express their ideas and opinions. Therefore, this program needs to be tested in clinical practice so that its viability can be confirmed. The experts were also consulted regarding the general use of the multimedia resources, slides for the sessions, and proposed items to include a variety of tools to ensure a better group dynamic, and most opinions were favorable.

Concerning the target population, people with mild and moderate depressive symptoms tend to respond well to most psychotherapy approaches, irrespective of whether those are self-help or cognitive-behavioral therapy (CBT) interventions (72–74). There are, however, reports of lower effects and high rates of treatment dropout when no human therapeutic support is offered, and the patient has only access to the content generated by the electronic platform (74, 75). There is clear evidence that the computer cannot fully replace human contact (76). Therefore, our intervention was developed as a synchronous online approach including full support of the healthcare professionals throughout the program for the participants, making it a relevant therapeutic alliance (73).

Finally, it is noteworthy that most studies investigating mood disorders and web-based treatments do not deal with major depression diagnoses, but with symptoms of depression (72, 77). Given the evidence found in the literature, our intervention was designed for individuals with depressive symptoms and not for those with a diagnosis of major depression. Most experts agreed that this intervention could be used for mild to moderate depressive symptoms, which is supported by the previous literature. Future studies using this protocol should explore which group of participants will benefit the most through this intervention.

The study has some limitations. First, the experts who evaluated the protocol were invited to participate through the authors' professional relationship network. Purposive sampling was used to recruit professionals to serve in the expert committee. However, experts were form different institutions, had different backgrounds, and experiences in the field. Second, the intervention was developed in Portuguese language. Although we had an English version translated by a professional native English speaker translator, no cross-cultural adaptation was carried out. This protocol should be tested in other languages and cultures to verify if the sessions are feasible and meaningful, aiming to make the flourishing intervention culturally sensitive.

## 5. Ethics and dissemination

The development of the flourishing intervention aims to fill the gap in public health approaches since it is a practical and low-cost intervention. Interventions in groups have an advantage of providing greater efficiency and cost-effectiveness to health services (78), besides the benefits to patients (79), as they facilitate the exchange of experiences and support by expanding the social support network.

Furthermore, the flourishing intervention aims to train healthcare professionals from different backgrounds and expertise, providing fundamental skills to conduct the intervention as a new healthcare tool. Investment in continuous education of multidisciplinary teams has proved to be a significant resource for implementing technical innovations, which aim to change practices in health systems and, consequently, in communities (80). The authors developed a guide for the providers on specific information for conducting the sessions, such as troubleshooting and managing therapeutic groups. The experts had access to this material and highlighted its relevance. The providers' guide is an accessible material for healthcare professionals to understand the exercises and tools proposed for the sessions. The intention is to allow this to be reproducible in different healthcare contexts. A study pointed out that it is important for professionals conducting online interventions to have clinical experience, empathy, and robust knowledge of the technique used, otherwise the intervention may have less effect (26). Therefore, adequate material and training are essential to ensure the best use and effectiveness of the intervention.

## 6. Conclusion

The development of the intervention based on the concept of flourishing obtained consensus from the experts. Necessary adjustments were made by the team, and the protocol reformulation proved to be successful. The intervention is ready to be tested in an experimental study to verify its feasibility and effectiveness.

## Data availability statement

The raw data supporting the conclusions of this article will be made available by the authors, without undue reservation.

## Ethics statement

The study was approved by the Research Ethics Committee of the School of Medicine of the University of Sao Paulo, Brazil, under approval number CAAE: 52554221.4.0000.0068. All respondents provided written informed consent. The patients/participants provided their written informed consent to participate in this study.

## Author contributions

JG: conception and design of the work, data collection, data analysis and interpretation, drafting the article, and final approval of the version to be published. CB: data collection, data analysis and interpretation, drafting the article, and final approval of the version to be published. WA: data analysis and interpretation, critical revision of the article, and final approval of the version to be published. CG: data interpretation, critical revision of the article, and final approval of the version to be published. GL: conception and design of the work, data analysis and interpretation, critical revision of the article, and final approval of the version to be published. HV: conception and design of the work, data interpretation, critical revision of the article, and final approval of the version to be published. All authors contributed to the article and approved the submitted version.
