# Supplementary figures and images for: Development of a comprehensive flourishing intervention to promote mental health using an e-Delphi technique
Source: Front Psychiatry. 2023 Feb 17;14:1064137. doi: 10.3389/fpsyt.2023.1064137 (PMC9981953; doi:10.3389/fpsyt.2023.1064137)

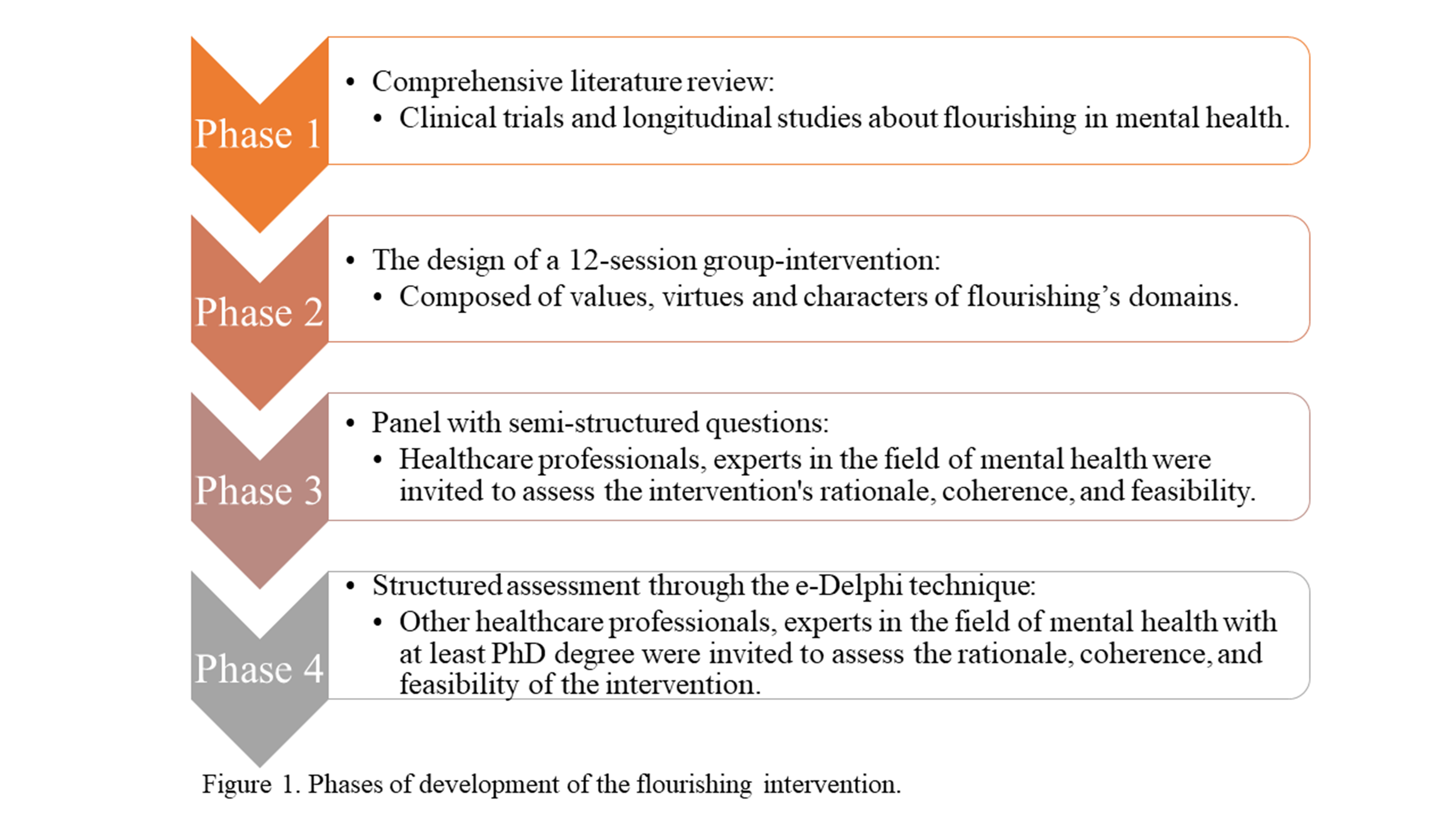

Supplement: Supplementary file 1 [file Image_1.TIF]
